# Experimental measurements of water molecule binding energies for the second and third solvation shells of [Ca(H_2_O)*_n_*]^2+^ complexes

**DOI:** 10.1098/rsos.160671

**Published:** 2017-01-04

**Authors:** E. Bruzzi, A. J. Stace

**Affiliations:** Department of Physical and Theoretical Chemistry, School of Chemistry, University of Nottingham, University Park, Nottingham NG7 2RD, UK

**Keywords:** calcium, water, binding energy, solvation shell

## Abstract

Further understanding of the biological role of the Ca^2+^ ion in an aqueous environment requires quantitative measurements of both the short- and long-range interactions experienced by the ion in an aqueous medium. Here, we present experimental measurements of binding energies for water molecules occupying the second and, quite possibly, the third solvation shell surrounding a central Ca^2+^ ion in [Ca(H_2_O)*_n_*]^2+^ complexes. Results for these large, previously inaccessible, complexes have come from the application of finite heat bath theory to kinetic energy measurements following unimolecular decay. Even at *n* = 20, the results show water molecules to be more strongly bound to Ca^2+^ than would be expected just from the presence of an extended network of hydrogen bonds. For *n* > 10, there is very good agreement between the experimental binding energies and recently published density functional theory calculations. Comparisons are made with similar data recorded for [Ca(NH_3_)*_n_*]^2+^ and [Ca(CH_3_OH)*_n_*]^2+^ complexes.

## 1. Introduction

Understanding the role played by Ca^2+^ in biological systems requires detailed knowledge of how the ion behaves in an aqueous environment [1]. Under circumstances where the binding energies of water molecules remain high it is very likely that, even with the rapid exchange of solvent molecules [2], Ca^2+^ will still have associated with it a significant number of water molecules that remain in close proximity to the central ion. Estimates of coordination numbers (CN) for the calcium ion in aqueous solution lie between 6 and 8; these values being derived primarily from diffraction experiments on concentrated solutions [1,3]. Two gas-phase studies by Bush *et al*. of the infrared spectroscopy of [Ca(H_2_O)*_n_*]^2+^ clusters, where *n* ranged from 4 to 69, have provided evidence of a transition in CN between the gaseous and condensed phase result when *n* ≥12 [4,5]. The coordination of Ca^2+^ in water has also been the subject of two recent extensive calculations where the authors have sought to identify the core structure in complexes containing up to 27 molecules [6,7]. Density functional theory (DFT) calculations by Lei and Pan showed no evidence for the emergence of a central [Ca(H_2_O)_8_]^2+^ core as the clusters increased in size [6]; however, they did observe temperature-dependent structural transitions in the second and third solvation shells. A temperature dependence was also recorded in the calculations of Bai *et al*. [7]; however, in their case it was the coordination number that changed, with low temperatures favouring 7 or 8, but switching to 6 as the temperature increased and facilitated the movement of molecules across low energy barriers separating different structures.

One area where significant insight into coordination can be derived is through the experimental determination of binding energies and how they vary according to the size and configuration of a complex. For metal dications, such as Ca^2+^, measurements of this nature in the gas phase can be difficult because of problems arising from charge transfer [8]; however, significant progress has been made in recent years and data are available for a number of metal dication complexes where the coordinating ligand is water [9–20]. In most instances, binding energy measurements on [M(H_2_O)*_n_*]^2+^ complexes are limited to values of *n* in the range 4–8; however, Peschke *et al.* [13] succeeded in extending the limit to 14 for several metal dications including Ca^2+^ coordinated with water. In two recent papers, it has been shown that the pick-up technique in conjunction with finite heat bath theory [21–25] can be used to extend both the size range over which ligand binding energies can be determined and type of the ligand under consideration [26,27]. Using this approach, results have recently been presented for the metal dications Mg^2+^, Ca^2+^ and Sr^2+^ in association with ammonia and methanol in complexes containing up to 20 molecules [26,27]. Presented here are new results for the system [Ca(H_2_O)*_n_*]^2+^ where, by taking measurements out as far as *n *= 20, it has been possible to characterize the influence a 2+ charge has on water molecules in the third solvation shell.

## 2. Experimental details

Detailed descriptions of the apparatus used for the generation, identification and detection of gas-phase multiply charged metal–ligand complexes have been given previously [28–30]. Briefly, mixed neutral clusters were generated by the adiabatic expansion of a water/argon gas mix through a pulsed supersonic nozzle at a backing pressure of between 1 and 5 bar. The resultant neutral clusters then passed through a region where calcium vapour (approximately 10^−2^ mbar) was generated by a Knudsen effusion cell (DCA Instruments, EC-40-63-21) operating at approximately 600°C. Neutral calcium atoms collided with the molecular cluster beam to produce various neutral clusters including some with the composition Ca(H_2_O)*_n_* and Ca.Ar*_m_*(H_2_O)*_n_*. Previous experiments have shown that argon atom evaporation is an essential part of the ‘pick-up’ process and facilitates the dispersion of energy on addition of a metal atom and after electron ionization [28–30]. Neutral clusters, some of which contain (on average) a single metal atom, enter the ion source of a high resolution, reverse geometry, double focusing mass spectrometer (VG-ZAB-E), where they were ionized by high-energy electron impact (approximately 70–100 eV). As only ions rather than neutral complexes are detected in the experiment, it is likely that extensive evaporation of ligands, predominantly argon, but also water molecules, takes place to reduce the internal energy of the complexes to a relatively stable level. The resulting ion beam was then extracted from the source at a potential voltage of 7 kV into the flight tube of a sector mass spectrometer and the mass-analysed ion kinetic energy (MIKE) technique used to study fragmentation occurring in the second field free region of the mass spectrometer between the magnetic and electric sector [31]. Each [Ca(H_2_O)*_n_*]^2+^ cluster dication was selected using the magnet and the electric sector field voltage was then scanned while the accelerating voltage and magnetic field remained constant. Equations given previously were then used to identify any fragment ions and determine their centre-of-mass kinetic energy [26,27], and for each of the complexes discussed here the principal fragmentation pathway observed was

2.1 $${\left[\text{Ca}{\left({\text{H}}_{2}\text{O}\right)}_{n}\right]}^{2+}\to {\left[\text{Ca}{\left({\text{H}}_{2}\text{O}\right)}_{n-1}\right]}^{2+}+{\text{H}}_{2}\text{O.}$$

**Figure 1.**
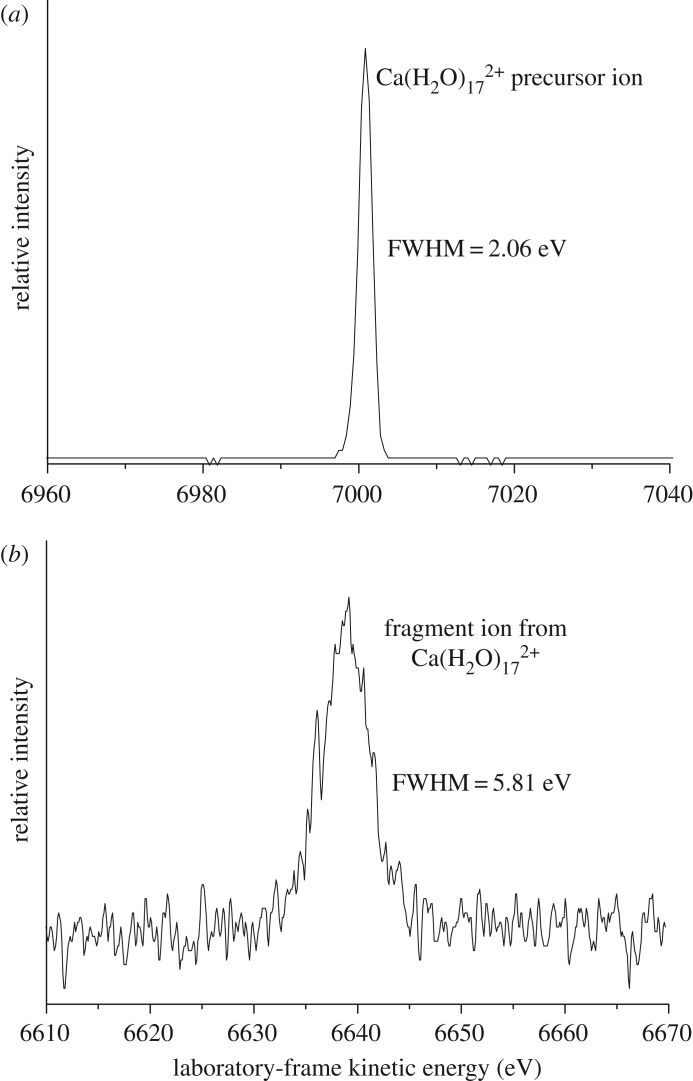
Examples of precursor (*a*) and fragment (*b*) peak profiles taken from the experimental results. The additional FWHM (full width half maximum) seen for the fragment ion is due the release of kinetic energy during the fragmentation process.

Figure 1 gives examples of precursor and fragment ion peak profiles, where the energy resolution of the mass spectrometer has been increased in order to minimize the energy width of the precursor ion while still maintaining a good signal-to-noise ratio for the fragment ion. The results of experimental measurements of the average kinetic energy released during reaction (2.1) are given are given in table 1 for *n* in the range 4–20; an earlier discussion on the stability of metal dication–water complexes noted the difficulty the pick-up technique has with generating calcium complexes, where *n* < 4 [32]. The upper limit of *n *= 20 is determined by a combination of declining signal strength and interference from artefact peaks [33]. Up to six measurements of kinetic energy release were made at each value of *n* and experimental uncertainties have been calculated from the spread in kinetic energy across the separate measurements.

**Table 1.** Summary of the experimental measurements of kinetic energy release (<$\varepsilon_{t}$>) and their uncertainties (±Δ<*ε*_t_>), together with binding energies (*E*_b_) and their uncertainties (±Δ*E*_b_) derived from finite heat bath theory.

*n*	<$\varepsilon_{t}$> (meV)	±Δ<$\varepsilon_{t}$> (meV)	*E*_b_ (kJ mol^−1^)	±Δ*E*_b_ (kJ mol^−1^)
4	38	5.3	125	17
5	37	5.1	98	13
6	30	5.0	70	11
7	30	7.4	65	16
8	30	2.9	62	6.0
9	28	2.6	55	5.1
10	25	3.8	49	7.2
11	26	3.8	48	7.0
12	24	3.5	44	6.4
13	24	1.3	43	2.4
14	26	2.4	46	4.3
15	23	3.4	40	5.9
16	22	1.8	38	3.2
17	24	3.4	41	5.9
18	28	2.4	48	4.1
19	23	2.7	40	4.5
20	22	1.2	38	2.1

## 3. Results and discussion

**Figure 2.**
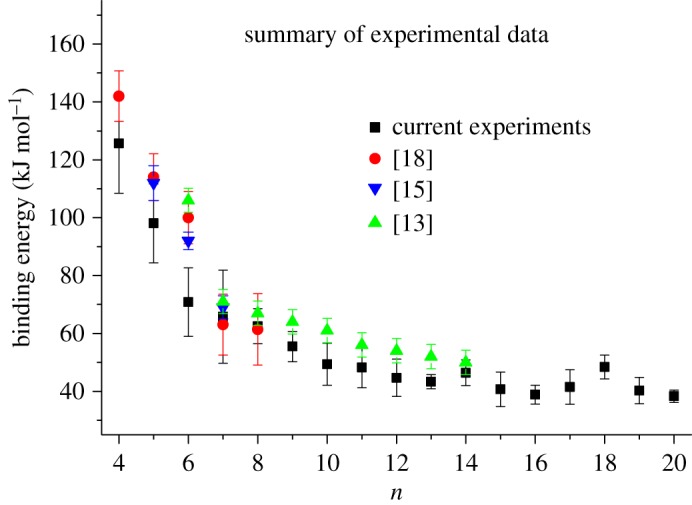
Comparisons between the binding energies derived from kinetic energy measurements and those recorded using alternative experimental techniques: Peschke *et al*. [13]—high pressure mass spectrometry; Rodriguez-Cruz *et al*. [15]—black-body infrared dissociation; Carl & Armentrout [18]—collision-induced dissociation.

Using finite heat bath theory [21–25], the kinetic energy measurements in table 1 have been transformed into binding energies for individual water molecules in [Ca(H_2_O)*_n_*]^2+^ complexes for *n* in the range 4–20. The assumptions made and the parameters specific for this particular series of experiments have been listed elsewhere [26,27]. The binding energies and their associated error limits are recorded in table 1 and plotted in figure 2 as a function of *n*. As noted in previous experiments, the error bars are largest for the smaller complexes (*n *≤ 7) because the fragment ion signals for these are weak which in turn makes for large inaccuracies. However, because the objective here is to extend the range of measurements out as far as the third solvation shell, it can be seen from figure 2 that this can be achieved with results that have comparatively small experimental errors. In addition, there are already a number of more accurate measurements from other groups on the smaller complexes and some of these are also plotted in figure 2 [13,15,18]. Overall, the binding energies reported here have slightly lower values than those recorded by other groups, but for the most part the error bars do overlap with those assigned to the existing data [13,15,18]. In addition to issues discussed above regarding signal levels for the smaller complexes, it is most probable that the ions studied in these experiments have higher internal temperatures than those generated at or close to 298 K [13,15,18]; however, an elevated temperature is more likely to result in a small increase in binding energy [34]. Where temperature could have an effect is in the case of the most obvious mismatch between these results and those recorded previously [13,15,18], namely [Ca(H_2_O)_6_]^2+^. In their measurements of hydration energies for alkaline earth metal ions, Rodriguez-Cruz *et al*. [15] provided evidence for the formation of a complex with the configuration [Mg(H_2_O)_5_(H_2_O)]^2+^, where the additional (H_2_O) denotes the presence of a water molecule that occupies an outer solvation shell and is hydrogen bonded to one or more molecules in the primary shell. This displacement of a water molecule was found to occur at an elevated temperature and was characterized by a lower than expected hydration enthalpy. Similar behaviour has been observed following kinetic energy release measurements on [Mg(NH_3_)*_n_*]^2+^ complexes [26], and formation of the isomers [Mg(NH_3_)_4_(NH_3_)]^2+^ and [Mg(NH_3_)_5_(NH_3_)]^2+^ (or [Mg(NH_3_)_4_(NH_3_)_2_]^2+^) was attributed to the low binding energies calculated from *finite heat bath* theory. Unlike the experiments of Rodriguez-Cruz *et al*. [15], it is not possible to adjust the internal temperature and so generate alternative isomeric forms in this study. Although similar behaviour was not observed for the complex [Ca(NH_3_)_6_]^2+^ [26], it is possible that the higher binding energies found here for outer-shell water molecules could stabilize alternative structure(s). Calculations by both Peschke *et al*. [13] and Bush *et al*. [4] place the [Ca(H_2_O)_5_(H_2_O)]^2+^ complex approximately 22 kJ mol^−1^ higher in energy than a ground state structure where all the water molecules are coordinated to the central ion; an internal energy of this magnitude is easily achieved during electron ionization.

**Figure 3.**
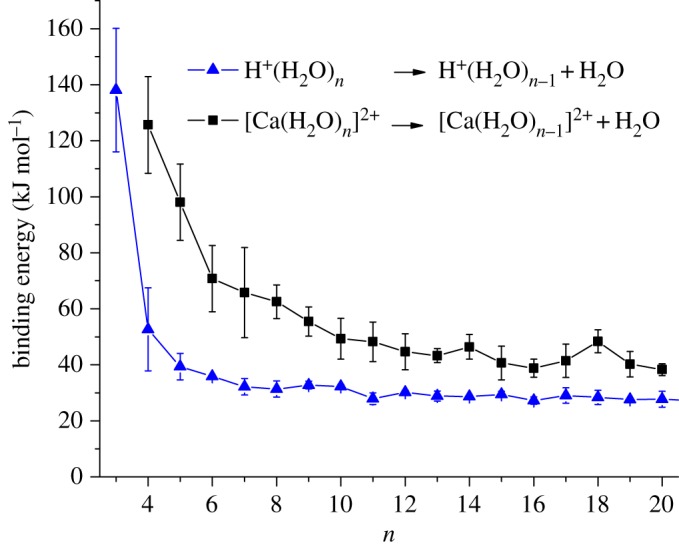
Comparison between experimental binding energies determined for [Ca(H_2_O)*_n_*]^2+^ and for H^+^(H_2_O)*_n_* plotted as a function of *n*. The data for H^+^(H_2_O)*_n_* have been adapted from [35].

As can be seen from figure 2, all of the datasets show a similar qualitative trend with a rapid drop in binding energy to *n* = 7 followed by a more gradual decline out to *n* = 20 in the case of the results presented here. To emphasize the decline in terms of the influence a 2+ charge has on water molecules in the larger complexes, figure 3 compares the binding energy data presented here with those recorded in earlier experiments on H^+^(H_2_O)*_n_* clusters [35], where it was found that, as *n* increased, the results rapidly converged to a value that was approximately equal to the strength of a single hydrogen bond. As can be seen from figure 3, the data for [Ca(H_2_O)*_n_*]^2+^ complexes show binding energies that, even for *n *= 20, remain high and will probably not converge close to those recorded for H^+^(H_2_O)*_n_* clusters (or the value for a hydrogen bond) until *n* is approximately 25 or more. In earlier discussions of the results derived from kinetic energy measurements, it has been argued that the nature of the experiment is such that only the lowest energy process available to a cluster will contribute to reaction (2.1) above [26,35]. A simple kinetic argument has also been presented in support of such a conclusion. That being the case, then similar behaviour is expected here and would be supported by numerous calculated examples of where low energy structures have outer-shell water molecules that are held in place by single acceptor bonds [6,7].

**Figure 4.**
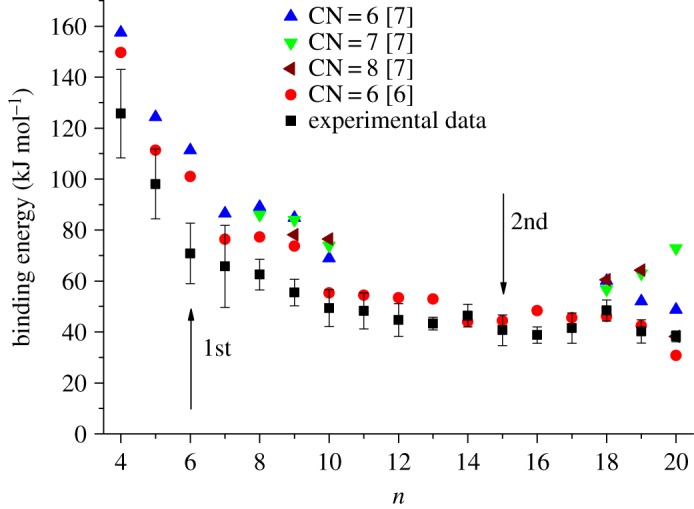
Comparison between experimental binding energies determined for [Ca(H_2_O)*_n_*]^2+^ and calculated results taken from Bai *et al*. [7] and Lei & Pan [6]. The data from Bai *et al*. [7] apply to Ca^2+^ coordination numbers (CN) lying between 6 and 8. The arrows denote the completion of solvation shells as identified from theory [6].

Lei & Pan [6] have undertaken calculations on the structural and spectroscopic properties of [Ca(H_2_O)*_n_*]^2+^ complexes and included in their results are sequential binding energies out to *n *= 20. Similarly, Bai *et al*. [7] have also presented binding energy data as part of a theoretical study into the static and dynamic properties of solvated Ca^2+^; their data cover a more limited size range, but do explore several options with regard to the core coordination number of Ca^2+^. Figure 4 compares the experimental binding energies with results taken from the calculations of Bai *et al*. [7] and Lei & Pan [6]. For complexes containing fewer than nine water molecules, the match with theory is closest for those experimental results taken from studies prior to this one [13,15,18]. However, for complexes containing 10 or more water molecules the agreement between the DFT results of Lei & Pan [6] when CN = 6 and the current experimental data is very good and, for the most part, within experimental error. It is interesting to note that the slight increase in experimental binding energy at *n *= 18 is reproduced by both sets of calculations [6,7]; however, beyond *n *= 18 it is again the CN = 6 data that most closely match the experimental results. As noted above, many of the calculated structures include low energy configurations where outer-shell water molecules are held in place with single acceptor hydrogen bonds, and these would be accessible at the elevated internal temperatures expected to be found in the complexes studied here. Likewise, the lower coordination number predicted by the calculations of Bai *et al*. [7] would again match our expectation for cluster ions generated by electron ionization. The arrows shown in figure 4 denote the sizes at which the first and second solvation shells surrounding Ca^2+^ are considered to be complete in the calculations of Lei & Pan [6]. That being the case, then our experimental measurements extend well into the third solvation shell and the comparison in figure 3 would suggest that the 2+ charge continues to have an influence on the binding of these outer water molecules to the central ion.

**Figure 5.**
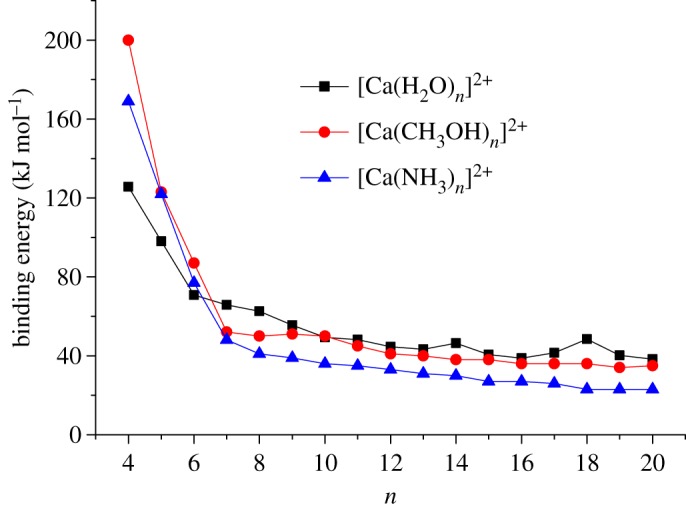
Comparison between the experimental data presented here for [Ca(H_2_O)*_n_*]^2+^ and results recorded previously for [Ca(NH_3_)*_n_*]^2+^ [26] and [Ca(CH_3_OH)*_n_*]^2+^ [27].

Finally, figure 5 makes a comparison between the data presented here for [Ca(H_2_O)*_n_*]^2+^ and results presented previously for [Ca(NH_3_)*_n_*]^2+^ and [Ca(CH_3_OH)*_n_*]^2+^ that have also been derived from experimental data using finite heat bath theory [26,27]. The error bars (not shown for purposes of clarity) on the results for *n* = 4 and 5 are probably too large for any meaningful interpretation; however, for both [Ca(NH_3_)*_n_*]^2+^ and [Ca(CH_3_OH)*_n_*]^2+^ it was concluded that the first solvation shell consists of six molecules and so it is interesting to see that all three datasets converge to approximately 80 kJ mol^−1^ at that point.

From *n* = 7, the decline in binding energy is, in all cases, very much less rapid than is seen for the small clusters and would suggest that this pattern follows a transition to the second solvation shell where binding energy is now determined by hydrogen bond strength, but one that appears enhanced by the presence of the 2+ charge on the metal. It is instructive to compare figure 5 with data recorded previously for the series (H_2_O)*_n_*H^+^, (CH_3_OH)*_n_*H^+^ and (NH_3_)*_n_*H^+^ [35], some of which are shown in figure 3. In the latter, the binding energies dropped abruptly until *n* = 6/7 and then exhibited no further decline, having reached values that match approximately hydrogen bond strengths found in neutral molecular pairs. The overall trend in binding energy being H_2_O ≈ CH_3_OH > NH_3_. The same ordering is seen in figure 5, but taking ammonia as an example, it can be seen that at *n* = 20 the measured binding energy is approximately 20 kJ mol^−1^, which is slightly higher than the hydrogen bond strength at 16 kJ mol^−1^. All three datasets show that the 2+ charge continues to influence molecular binding energies out into the third solvation shell. With regard to [Ca(H_2_O)*_n_*]^2+^ complexes, a similar conclusion on the long-range influence of charge was reached by Bush *et al*. [5] from their studies of infrared action spectra as a function of *n*. A comparable study by Walters *et al*. [36], but this time on singly charged Ni^+^(H_2_O)*_n_* complexes, provided evidence of the charge controlling the development of a hydrogen bond network in the second solvation shell. These studies show that the simple idea of ion solvation where the charge on a metal cation is contained and accommodated by a single shell of solvent molecules is no longer an adequate description of events taking place in solution.

## 4. Conclusion

The application of finite heat bath theory to kinetic energy release measurements recorded in the gas phase on [Ca(H_2_O)*_n_*]^2+^ complexes has made it possible to extract binding energies for up to 20 water molecules bound to Ca^2+^. The results suggest that the first solvation shell contains six water molecules; however, more significant is the observation that the 2+ charge on the metal cation has an influence on molecular interactions that extends far beyond the first solvation shell.

## Data accessibility

A complete dataset of measurements is available from Dryad: http://dx.doi.org/10.5061/dryad.fv4b5 [37].
